# The Potential of *Flos sophorae immaturus* as a Pigment-Stabilizer to Improve the *Monascus* Pigments Preservation, Flavor Profiles, and Sensory Characteristic of *Hong Qu* Huangjiu

**DOI:** 10.3389/fmicb.2021.678903

**Published:** 2021-05-20

**Authors:** Yijin Yang, Yongjun Xia, Xin Song, Zhiyong Mu, Huazhen Qiu, Leren Tao, Lianzhong Ai

**Affiliations:** ^1^Shanghai Engineering Research Center of Food Microbiology, School of Medical Instrument and Food Engineering, University of Shanghai for Science and Technology, Shanghai, China; ^2^School of Energy and Power Engineering, University of Shanghai for Science and Technology, Shanghai, China

**Keywords:** Hong Qu Huangjiu, *Flos sophorae immaturus*, *Monascus* pigments, flavor profiles, sensory characteristic

## Abstract

Hong Qu Huangjiu (HQW) is distinguished by its inclusion of *Monascus* pigments, meaning that photosensitivity strongly affects the sensory quality of the wine. In this study, the effects of *Flos sophorae immaturus* (*FSI*) on the stability of *Monascus* pigments, the flavor profiles, and the sensory characteristics of HQW were investigated. After sterilization, the addition of *FSI* increased the preservation rate of *Monascus* pigments in HQW by up to 93.20%, which could be accounted for by the synergy of rutin and quercetin in *FSI*. The total content of the volatile flavor compounds in HQW increased significantly as the added amounts of *FSI* were increased, especially 3-methyl-1-butanol, 2-methyl-1-propanol, and short-chain fatty acid ethyl esters (SCFAEE). Sensory evaluation and partial least-squares regression revealed that the concentration of *FSI* significantly affected the aroma characteristics of HQW but had little effect on the mouthfeel. The addition of 0.9 mg/mL *FSI* yielded a satisfactory HQW with high scores in terms of mouthfeel and aroma. The strong correlation between fruit-aroma, full-body, and SCFAEE suggests that *FSI* might alter the aroma of HQW by enhancing the synthesis of SCFAEE. Summarily, treatment with *FSI* represents a new strategy for improving the stability of photosensitive pigments and thus adjusting the aroma of HQW or similar beverages.

## Introduction

Hong Qu Huangjiu (HQW), a typical form of Huangjiu (Chinese rice wine), has become increasingly popular in the rice wine market in recent years (Huang et al., 2019; Wang et al., 2020). Consumers favor HQW because of its bright red color, fine flavor, and health-promoting aspects (Park et al., 2016). The active ingredients of HQW have been proven to reduce blood pressure and blood lipid and cholesterol concentrations, and to influence metabolism and immune regulation in the human body (Lin et al., 2017; Mondal et al., 2019). Accordingly, HQW is referred to as a “ruby” Huangjiu wine.

Hong Qu Huangjiu is unique because its production involves Hong Qu (saccharification starter), which is inoculated with *Monascus* spp., introducing numerous pigments and enzymes (Feng et al., 2012; Patakova, 2013; Vendruscolo et al., 2016; Keekan et al., 2020). *Monascus* pigments contribute to the unique color and physiological effects of HQW. However, as a natural pigment, *Monascus* pigments undergo substantial photodegradation during storage, which negatively affects the appearance, sensory profile, and commercial value of HQW (Mapari et al., 2009; Wang et al., 2016; Sen et al., 2019). Although storage in brown bottles can reduce the degradation of *Monascus* pigments, these do not fully prevent the color fading of HQW during storage. To resolve this problem, manufacturers prefer the use of caramel coloring to mask the photodegradation of *Monascus* pigments. However, caramel coloring alters the original color of HQW and may have negative effects on food safety and human health (Vollmuth, 2018; Mateo-Fernández et al., 2019). Thus, the effective and inert stabilizers of *Monascus* pigments are needed to reduce the photodegradation of HQW during storage, and thus maintain and improve its sensory profile.

Numerous studies have reported that antioxidants effectively prevent the photodegradation of *Monascus* pigments (Dhale et al., 2018; Liu C. et al., 2020). Therefore, antioxidants may improve the final sensory profiles of HQW. However, the application of traditional food-industry antioxidants (e.g., Vitamin C, quercetin, and β-carotene) is limited by high cost or poor water-solubility (Qian et al., 2012; Horuz et al., 2017). Therefore, inexpensive natural herbs that contain abundant antioxidant compounds may be effective alternatives for reducing the photodegradation of *Monascus* pigments in HQW.

*Flos sophorae immaturus* (*FSI*) is the dried flower bud of *Sophora japonic*a L. This herb has a high flavonoid content and is considered a healthy food because of its high antioxidant activity (Abdelhady et al., 2015; Li and Fan, 2020). *FSI* has been widely used for 2,000 years in China as an important traditional Chinese medicine, due to its antitumor, anti-inflammatory, and antibacterial activities (Liu T. et al., 2019; Huang et al., 2020). Many studies have reported that the antioxidant compounds of *FSI* are responsible for its efficacy (Yao et al., 2011; Zhang et al., 2011; Hu et al., 2016; Wang et al., 2019). Therefore, *FSI* may prevent the photodegradation of *Monascus* pigments in HQW during storage.

In our previous study (submitted for publication), we observed that *FSI* preserved *Monascus* pigments to a greater extent than honeysuckle (a natural herb with high antioxidant activity) and some traditional antioxidants (e.g., Vc, EDTA, β-cyclodextrin, and quercetin). Thus, this study aimed to analyze the ability of *FSI* to preserve *Monascus* pigments in HQW during different brewing stages. Specifically, we evaluated the effects of *FSI* on the oenological properties, flavor profiles, and sensory characteristics in the resulting HQW. This study provides a basis for improving the pigment stability and sensory quality of HQW and broadens the applications for *FSI* as a novel stabilizer in beverages containing photosensitive pigments.

## Materials and Methods

### Materials

Hong Qu (Saccharification starter) made using *Monascus* spp. was purchased from a commercial production facility in Gutian, Fujian province, China. *FSI* was picked from Linyi, Shandong province, China. Chlorogenic acid, rutin, and quercetin were purchased from Shanghai Yuanye Bio-Technology Co., Ltd. (Shanghai, China). All reagents and standards were purchased at Sigma Aldrich (Shanghai, China). n-Alkane (C_7_-C_40_; Supelco, Bellefonte, PA, United States) standards were used to determine the retention index.

Ten grams of Hong Qu was added in 1 L of 15% ethanol, ultrasonicated for 20 min at room temperature, and filtrated to obtain the *Monascus* pigments-solution. To obtain an extract, fresh *FSI* was dehydrated and dried in an oven at 40°C to a constant level of moisture. The dry *FSI* was then thoroughly milled using a grinder to produce powder, which was passed through a 60-mesh sieve. Two grams of the sieved powder were mixed with 40 mL of 40% ethanol at 70°C, ultrasonicated for 20 min, and filtered immediately. The filtrate was pooled and concentrated using a rotary evaporator at 55°C. Finally, a dry powder was obtained by freeze drying and stored in a desiccator in the dark at 4°C.

### The Preservation Rate of *Monascus* Pigments

Ultraviolet (UV) was used to accelerate the degradation of *Monascus* pigments-solution. Chlorogenic acid, rutin, quercetin, and *FSI* were, respectively, mixed with *Monascus* pigments-solution to prepare solutions at a concentration of 0.5 mg/mL. A volume of 50 mL mixed solution was placed in a UV-transparent quartz dish with a radius of 8 cm for UV irradiation, while the pure *Monascus* pigments-solution was set as the control. The maximum absorbance (A_max_) of *Monascus* pigments-solution and HQW were determined using a SpectraMax M5e spectrofluorometer (Molecular Devices Ltd., San Jose, CA, United States). The *Monascus* pigments-preservation rate was calculated using the following formula:

$$\mathrm{Preservation}\mathrm{rate}\mathrm{of}\mathrm{pigment}\left(\%\right)=A_{x}/A_{o}\times 100\%$$

where A_x_ and A_0_ are the A_max_ values of pigments treated and untreated with *FSI*, respectively.

### Antioxidant Constituent of *FSI*

The possibly antioxidant constituent of *FSI* was analyzed using a Waters high-performance liquid chromatograph (HPLC) system (e2695-Empower system, MA, United States). An equivalent of 5.0 mg of *FSI* was solubilized in 25 mL methanol, filtered through a 0.22 μm filter membrane, and separated on a Sepax HP-C18 column (4.6 mm × 250 mm, 5 μm) at 25°C. The mobile phase was composed of 100% acetonitrile/0.01 mol/L ammonium acetate (0 min, 10:90 and 30 min, 30:70). The flow rate was 1.0 mL/min, the injection volume was 10 μL, and the UV detection wavelength was set at 350 nm.

### Brewing of HQW

Hong Qu Huangjiu was brewed according to the method described by Yang et al. (2017). *FSI* was correspondingly added during Hong Qu soaking, on day 5 of fermentation (representative of the fermentation stage), and cooled after sterilization. The *FSI* concentration in the wine was adjusted to 0, 0.1, 0.3, 0.5, 0.7, and 0.9 mg/mL. As the photodegradation of *Monascus* pigments in HQW generally occurs during storage, the young wine was stored in the dark at 4°C for 160 days, and changes were monitored. The aged wine was regularly sampled, and samples were stored at −80°C for further analysis.

### Oenological Properties of HQW

Reducing sugar was detected using the method described by Miller (1959). Total acidity was determined according to Code of China Standard No. GB/T 5517-2010 (GB/T 5517-2010), and ethanol was determined using the HPLC method reported by Di Egidio et al. (2010), with the following conditions: a Carbomix H-NP (300 mm × 7.8 mm) column at a temperature of 55°C, a mobile phase of 2.5 mM sulfuric acid at a flow rate of 0.6 mL/min and using a differential detector. The amino acid nitrogen content in a 5-mL aliquot of HQW digested at 420 °C for 150 min was determined using an automatic Kjeldahl apparatus (Kjeltec 8400, FOSS Co., Sweden).

### Analysis of Volatile Flavor Compounds

Volatile flavor components were extracted from HQW by solid-phase microextraction and analyzed by gas chromatography–mass spectrometry (GC-MS), according to the method reported by Yang et al. (2019). A 5-mL aliquot of each sample [diluted to 6% (v/v) ethanol] was placed in a 20-mL headspace glass vials with 2 g of sodium chloride and 10 μL internal standard of 4-methyl-2-pentanol (250 μg/mL). The volatile compounds were extracted for 30 min at 50°C using a 50 μm DVB/CAR/PDMS fiber. Then, the extracted fiber head was desorbed at the GC injection port at 250°C for 7 min for GC-MS analysis. The chromatographic conditions of GC-MS system (Thermo Fisher Inc., United States) equipped with a DB-WAX column (60 m × 0.25 mm × 0.25 μm, Agilent Technologies, United States) were as follows: programmed at an initial temperature of 40°C for 3 min, increased to 210°C at 6°C/min, and then, at 8°C/min to 230°C that is maintained for 15 min. Helium was delivered at a flow rate of 1 mL/min as the carrier gas. The conditions of the mass detector and identification of volatile compounds were operated as the method described by Yang et al. (2019). The quantity of volatile compounds was determined by the internal standard method.

### Sensory Evaluation

The sensory evaluation team comprised 10 judges (five men and five women) who had been trained to evaluate the sensory attributes of HQW samples using a series of reference substances, according to the ISO Standard No. 8586-1 (ISO, 1993; Supplementary Table 1). After the judges reached a consensus on the sensory characteristics of all substances, they were given a sensory assessment card that listed all attributes with their definitions and 10-point scales (from 0 to 9). The scoring criteria were as follows: 0 meant very weak, 1–2 means weak, 3–4 meant average, 6 meant medium, 7–8 means high, and 9 meant extremely high. The evaluation was conducted in a room with a uniform light source and without noise or other distracting stimuli. The HQW samples were presented to the judges in a random order, in marked tulip-shaped glasses.

### Statistical Analysis

Each sample was analyzed in triplicate, and the results were expressed as mean values ± standard deviations (SD). Duncan’s test was used to analyze the significant differences between data using SPSS 19.0 software (SPSS Inc., Chicago, IL, United States). Partial least squares regression (PLSR) was used to explore the relationship among volatile flavor compounds, sensory attributes, and HQW samples containing different concentrations of *FSI* using UNSCRAMBLER ver. 9.7 (CAMO ASA, Oslo, Norway). TBtools (Chen et al., 2020) were used to visualize the differentiation of flavor compounds in different wines.

## Results

### Effect of Adding *FSI* During Different Brewing Stages on the Preservation of *Monascus* Pigments in HQW

**FIGURE 1:**
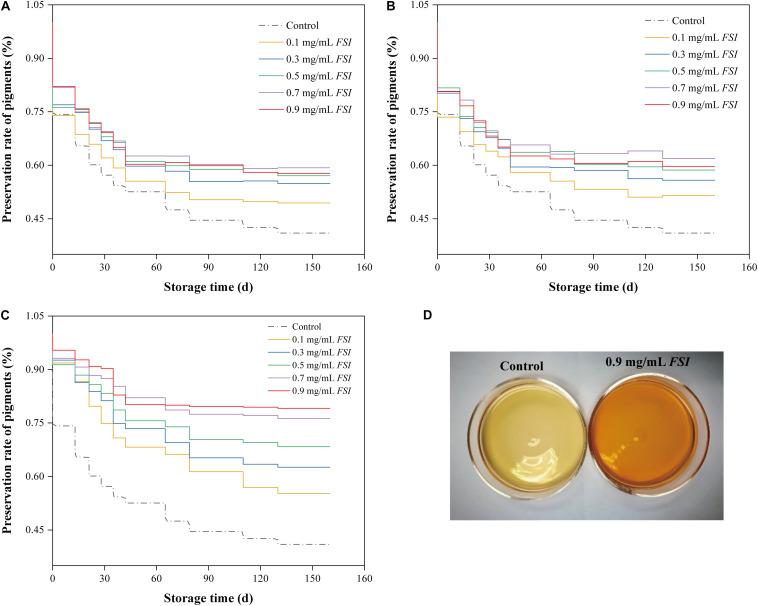
The preservation rate of *Monascus* pigments in HQW treated with different concentrations of *FSI* during Hong Qu soaking **(A)**, wine fermentation **(B)**, and after sterilization **(C)** during 160-days storage. Appearance comparison between HQW containing 0.9 mg/mL *FSI* and the control HQW **(D)**.

Photosensitive *Monascus* pigments may be degraded during the Hong Qu soaking stage of the brewing processes. Therefore, we investigated the preservation rates of *Monascus* pigments in HQW samples treated with different concentrations of *FSI* during different brewing stages (soaking, fermentation, and sterilization) (Figure 1). We found that the pigment-preservation rate increased with *FSI* concentration. Specifically, when 0.1–0.9 mg/mL *FSI* was added during Hong Qu soaking, the *Monascus* pigment-preservation rates increased by 20.68, 34.01, 39.33, 44.75, and 40.82% relative to the control (Figure 1A). When *FSI* was added during HQW fermentation, the pigment-preservation rate increased by 25.74, 36.25, 43.16, 58.46, and 53.02% relative to the control (Figure 1B). When *FSI* was added after sterilization, the rate increased by 34.89, 52.88, 66.87, 86.13, and 93.20% relative to the control (Figure 1C). After sterilization, the addition of 0.9 mg/mL *FSI* resulted in the greatest preservation rate of *Monascus* pigments. A similar pattern was observed for the appearance of HQW (Figure 1D). After natural aging for 160 d, the color of the control HQW had faded distinctly, whereas that of *FSI*-treated HQW remained satisfactory.

### Major Antioxidant Constituents in *FSI*

**FIGURE 2:**
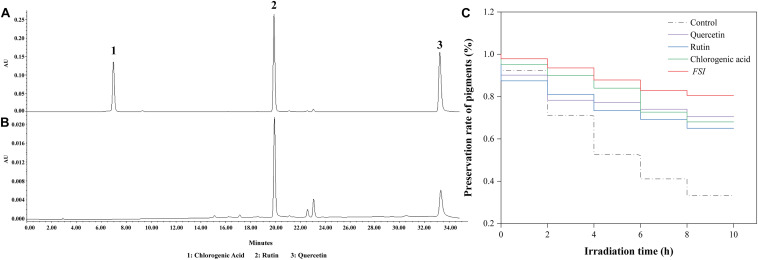
Effective constituent of *FSI* in the preservation of *Monascus* pigments. HPLC results of reference substances **(A)** and *FSI* **(B)**. Effect of quercetin, rutin, and *FSI* on the preservation rate of *Monascus* pigments **(C)**. Component 1: chlorogenic acid; Component 2: rutin; Component 3: quercetin.

The effective antioxidant compositions in *FSI* were analyzed using HPLC, with rutin, chlorogenic acid, and quercetin were used as references (Figure 2A). The result showed that *FSI* contained abundant rutin and quercetin and their contents were 68.64 and 23.75 mg/g, respectively (Figure 2B). Furthermore, the effects of rutin, quercetin, and *FSI* on the preservation rate of *Monascus* pigments under UV were compared (Figure 2C). After irradiation by UV for 10 h, the preservation rate of *Monascus* pigments in the control, solutions containing *FSI*, rutin, and quercetin correspondingly decreased from 100 to 33.2, 80.58, 68.05, and 70.53%. *FSI* exhibited better ability in the preservation of *Monascus* pigments than rutin or quercetin.

### Effect of Adding *FSI* During Different Brewing Stages on the Oenological Properties of HQW

**FIGURE 3:**
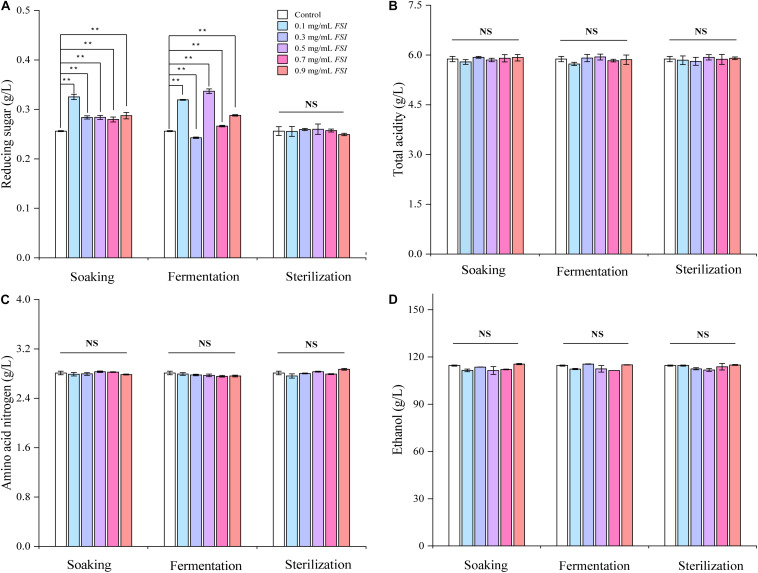
The oenological properties of HQW treated with various concentrations of *FSI* during different brewing stages, comprising reducing sugar content **(A)**, total acidity **(B)**, amino acid nitrogen content **(C)**, and ethanol content **(D)**. **Indicates significant differences (*p* < 0.01, Duncan’s tests) between different HQW. NS indicates no significant difference between the wine samples.

We next evaluated the oenological properties of HQW treated with *FSI* during different brewing stages (Figure 3). When *FSI* was added during Hong Qu soaking or fermentation, the reducing sugar content in the resultant HQW increased significantly. In contrast, adding *FSI* after sterilization had little effect on this parameter (Figure 3A). Otherwise, the addition of *FSI* during different brewing stages had little effect on the ethanol, total acidity, and amino-acid nitrogen contents in the final wine samples (Figures 3B–D). Because the addition of *FSI* after sterilization had little effect on the oenological properties and yielding the best protection against *Monascus* pigments loss, only wine samples to which *FSI* had been added after sterilization were further analyzed.

### Effect of Adding *FSI* After Sterilization on the Flavor Profiles of HQW

**FIGURE 4:**
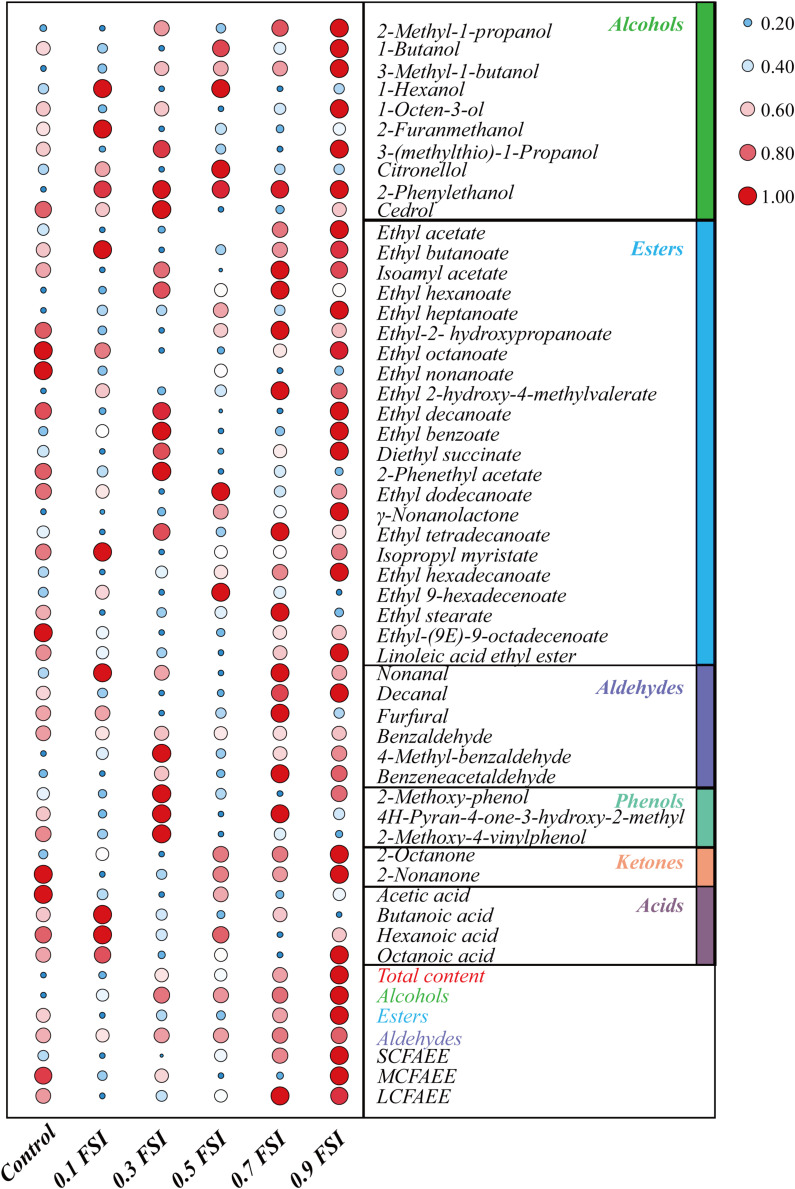
Differentiation of volatile flavor compounds in HQW containing different concentrations of *FSI*. The 0.1 *FSI*, 0.3 *FSI*, 0.5 *FSI*, 0.7 *FSI*, and 0.9 *FSI* represented HQW containing 0.1, 0.3. 0.5, 0.7, and 0.9 mg/mL *FSI*, respectively. The color and size of the circle represent the relative content of the flavor compound (the data has been standardized). The redder (the bigger) the circle, the higher the content of the flavor compound, and the bluer (the smaller) the circle, the lower content of the flavor compound.

The volatile flavor compounds of aged HQW treated with *FSI* after sterilization were analyzed (Table 1). A total of 47 compounds were detected, comprising 10 alcohols, 22 esters, six aldehydes, three phenols, two ketones, and four acids. The *FSI* had little effect on the detected number of flavor compounds, whereas the relative contents of flavor compounds were different in HQW samples containing different concentrations of *FSI* (Figure 4). In particular, the total content of volatile flavor compounds in HQW increased significantly as the added amounts of *FSI* were increased. HQW containing 0.9 mg/mL *FSI* exhibited the highest flavor-compound content (98.72 mg/L), followed by HQW containing 0.7 mg/mL *FSI* (95.61 mg/L), which were 10.64 and 7.15% higher than the control HQW, respectively (Table 1).

**TABLE 1:** Identification and relative contents of volatile flavor compounds in aged HQW (160 d) containing with different concentrations of *FSI*.

Code	Compounds	KI	Identification	Relative content (mg/L)					
				
				Control	0.1 *FSI*	0.3 *FSI*	0.5 *FSI*	0.7 *FSI*	0.9 *FSI*
**Alcohols**									
al1	2-Methyl-1-propanol	1,095	MS,RI	1.81 ± 0.04^b^	1.68 ± 0.05^a^	2.39 ± 0.02^d^	1.94 ± 0.08^c^	2.55 ± 0.03^e^	2.76 ± 0.06^f^
al2	1-Butanol	1,158	MS,RI	0.09 ± 0.001^a^	0.07 ± 0.005^a^	0.05 ± 0.003^a^	0.11 ± 0.006^b^	0.08 ± 0.009^a^	0.12 ± 0.002^b^
al3	3-Methyl-1-butanol	1,212	MS,RI	32.81 ± 0.47^a^	33.86 ± 0.39^b^	35.61 ± 0.51^c^	35.75 ± 0.32^c^	35.87 ± 0.36^c^	37.4 ± 0.44^d^
al4	1-Hexanol	1,345	MS,RI	0.04 ± 0.001^a^	0.06 ± 0.002^b^	0.03 ± 0.001^a^	0.06 ± 0.002^b^	0.03 ± 0.001^a^	0.04 ± 0.001^a^
al5	1-Octen-3-ol	1,456	MS,RI	0.09 ± 0.002^a^	0.07 ± 0.001^a^	0.09 ± 0.002^a^	0.06 ± 0.001^a^	0.08 ± 0.003^a^	0.11 ± 0.003^b^
al6	2-Furanmethanol	1,661	MS,RIL	0.19 ± 0.002^b^	0.24 ± 0.004^c^	0.13 ± 0.006^a^	0.17 ± 0.004^b^	0.15 ± 0.001^a^	0.18 ± 0.003^b^
al7	3-Methylthiopropanol	1,722	MS,RIL	0.91 ± 0.01^c^	0.83 ± 0.007^a^	0.96 ± 0.09^d^	0.86 ± 0.03^b^	0.81 ± 0.004^a^	0.98 ± 0.02^d^
al8	Citronellol	1,772	MS,RIL	0.02 ± 0.001^a^	0.03 ± 0.001^a^	0.01 ± 0.001^a^	0.04 ± 0.001^a^	0.02 ± 0.001^a^	0.02 ± 0.001^a^
al9	2-Phenylethanol	1,876	MS,RI	29.85 ± 0.67^a^	33.12 ± 0.83^b^	33.51 ± 0.91^b^	33.27 ± 0.75^b^	33.38 ± 0.66^b^	33.55 ± 0.72^b^
al10	Cedrol	2,105	MS,RIL	0.09 ± 0.001^b^	0.08 ± 0.003^b^	0.1 ± 0.002^b^	0.05 ± 0.002^a^	0.06 ± 0.001^a^	0.08 ± 0.001^b^
Total				54.00	59.29	60.44	60.26	60.4	62.36
**Esters**									
et1	Ethyl acetate	837	MS,RI	2.34 ± 0.05^c^	1.99 ± 0.06^a^	2.12 ± 0.09^b^	2.48 ± 0.04^d^	2.69 ± 0.05^e^	2.95 ± 0.06^f^
et2	Ethyl butanoate	1,049	MS,RI	0.72 ± 0.007^b^	0.76 ± 0.008^b^	0.66 ± 0.008^a^	0.69 ± 0.01^a^	0.73 ± 0.005^b^	0.75 ± 0.008^b^
et3	Isoamyl acetate	1,135	MS,RI	1.08 ± 0.01^b^	0.91 ± 0.007^a^	1.11 ± 0.01^b^	0.92 ± 0.004^a^	1.17 ± 0.04^c^	1.13 ± 0.03^c^
et4	Ethyl hexanoate	1,242	MS,RI	0.03 ± 0.001^a^	0.04 ± 0.001^a^	0.08 ± 0.003^b^	0.06 ± 0.002^a^	0.09 ± 0.001^b^	0.06 ± 0.002^a^
et5	Ethyl heptanoate	1,318	MS,RI	0.01 ± 0.001^a^	0.02 ± 0.003^a^	0.02 ± 0.002^a^	0.03 ± 0.001^a^	0.02 ± 0.001^a^	0.04 ± 0.001^a^
et6	Ethyl-2- hydroxypropanoate	1,346	MS,RI	1.69 ± 0.08^d^	1.51 ± 0.02^c^	1.44 ± 0.05^a^	1.62 ± 0.04^c^	1.75 ± 0.06^d^	1.63 ± 0.04^c^
et7	Ethyl octanoate	1,440	MS,RI	1.35 ± 0.02^d^	1.26 ± 0.06^c^	1.01 ± 0.01^a^	1.06 ± 0.03^a^	1.19 ± 0.05^b^	1.33 ± 0.02^d^
et8	Ethyl nonanoate	1,528	MS,RI	0.12 ± 0.003^b^	0.09 ± 0.006^a^	0.11 ± 0.001^b^	0.1 ± 0.005^b^	0.08 ± 0.008^a^	0.09 ± 0.002^a^
et9	Ethyl 2-hydroxy-4-methylvalerate	1,578	MS,RIL	0.02 ± 0.001^a^	0.05 ± 0.003^b^	0.03 ± 0.001^a^	0.04 ± 0.002^a^	0.07 ± 0.001^b^	0.06 ± 0.001^b^
et10	Ethyl decanoate	1,640	MS,RI	1.59 ± 0.07^c^	1.22 ± 0.09^b^	1.63 ± 0.03^d^	1.17 ± 0.006^a^	1.15 ± 0.04^a^	1.68 ± 0.06^d^
et11	Ethyl benzoate	1,675	MS,RI	0.95 ± 0.007^a^	0.96 ± 0.008^a^	0.98 ± 0.01^a^	0.94 ± 0.02^a^	0.95 ± 0.007^a^	0.98 ± 0.02^a^
et12	Diethyl succinate	1,688	MS,RI	0.53 ± 0.003^b^	0.42 ± 0.007^a^	0.66 ± 0.005^c^	0.45 ± 0.006^a^	0.57 ± 0.003^b^	0.71 ± 0.008^d^
et13	2-Phenethyl acetate	1,821	MS,RI	0.58 ± 0.005^b^	0.49 ± 0.004^a^	0.62 ± 0.007^b^	0.42 ± 0.004^a^	0.5 ± 0.008^a^	0.46 ± 0.005^a^
et14	Ethyl dodecanoate	1,837	MS,RI	0.58 ± 0.006^c^	0.55 ± 0.004^b^	0.48 ± 0.009^a^	0.61 ± 0.004^d^	0.53 ± 0.003^b^	0.57 ± 0.006^c^
et15	γ-Nonanolactone	2,011	MS, RI	0.02 ± 0.001^a^	0.03 ± 0.002^a^	0.05 ± 0.002^a^	0.12 ± 0.005^c^	0.09 ± 0.003^b^	0.17 ± 0.006^d^
et16	Ethyl tetradecanoate	2,036	MS,RI	1.95 ± 0.09^b^	1.84 ± 0.07^a^	2.05 ± 0.1^c^	1.91 ± 0.04^b^	2.09 ± 0.09^c^	1.98 ± 0.06^b^
et17	Isopropyl myristate	2,041	MS,RI	0.08 ± 0.002^b^	0.09 ± 0.003^b^	0.05 ± 0.002^a^	0.07 ± 0.001^a^	0.07 ± 0.001^a^	0.08 ± 0.003^b^
et18	Ethyl hexadecanoate	2,258	MS,RI	7.31 ± 0.21^b^	7.1 ± 0.16^a^	7.38 ± 0.27^b^	7.45 ± 0.13^c^	7.56 ± 0.18^d^	7.74 ± 0.15^e^
et19	Ethyl 9-hexadecenoate	2,270	MS,RIL	0.11 ± 0.007^a^	0.13 ± 0.005^b^	0.09 ± 0.004^a^	0.16 ± 0.008^c^	0.12 ± 0.009b	0.09 ± 0.004^a^
et20	Ethyl stearate	2,454	MS,RI	0.42 ± 0.005^c^	0.33 ± 0.008^a^	0.37 ± 0.008^b^	0.39 ± 0.004^b^	0.47 ± 0.006^d^	0.36 ± 0.005^a^
et21	Ethyl (9E)-9-octadecenoate	2,475	MS,RIL	0.95 ± 0.02^c^	0.84 ± 0.01^b^	0.75 ± 0.009^a^	0.79 ± 0.01^a^	0.86 ± 0.007^b^	0.87 ± 0.005^b^
et22	Linoleic acid ethyl ester	2,513	MS,RIL	1.38 ± 0.02^c^	1.31 ± 0.01^b^	1.27 ± 0.04^b^	1.19 ± 0.03^a^	1.35 ± 0.05^b^	1.46 ± 0.07^d^
Total				23.81	21.94	22.96	22.67	24.10	24.99
**Aldehydes**									
ad1	Nonanal	1,390	MS,RI	0.03 ± 0.001^a^	0.05 ± 0.002a	0.04 ± 0.002^a^	0.02 ± 0.001^a^	0.05 ± 0.001^a^	0.04 ± 0.001^a^
ad2	Furfural	1,442	MS,RI	0.39 ± 0.01^a^	0.39 ± 0.009^a^	0.37 ± 0.007^a^	0.38 ± 0.005^a^	0.4 ± 0.008^a^	0.38 ± 0.009^a^
ad3	Decanal	1,503	MS,RIL	0.06 ± 0.002^b^	0.04 ± 0.001^a^	0.02 ± 0.001^a^	0.03 ± 0.001^a^	0.08 ± 0.003^b^	0.09 ± 0.003^b^
ad4	Benzaldehyde	1,518	MS,RI	3.56 ± 0.17^c^	3.38 ± 0.15^a^	3.49 ± 0.20^b^	3.55 ± 0.12^c^	3.41 ± 0.16^a^	3.49 ± 0.14^b^
ad5	4-Methyl-benzaldehyde	1,644	MS,RI	0.28 ± 0.03^a^	0.31 ± 0.01^a^	0.35 ± 0.009^b^	0.3 ± 0.005^a^	0.32 ± 0.009^b^	0.33 ± 0.01^b^
ad6	Benzeneacetaldehyde	1,654	MS,RI	0.39 ± 0.006^a^	0.36 ± 0.005^a^	0.45 ± 0.02^b^	0.39 ± 0.007^a^	0.51 ± 0.005^c^	0.48 ± 0.008^b^
Total				4.71	4.53	4.72	4.67	4.77	4.81
**Phenols**									
ph1	2-Methoxy-phenol	1,866	MS,RI	0.26 ± 0.01^b^	0.24 ± 0.04^a^	0.31 ± 0.03^c^	0.25 ± 0.009^b^	0.22 ± 0.02^a^	0.29 ± 0.01^c^
ph2	4H-Pyran-4-one, 3-hydroxy-2-methyl	1,970	MS,RIL	0.06 ± 0.003^b^	0.04 ± 0.001^a^	0.08 ± 0.002^b^	0.03 ± 0.001^a^	0.08 ± 0.003^b^	0.05 ± 0.002^a^
ph3	2-Methoxy-4-vinylphenol	2,215	MS,RIL	0.19 ± 0.008^b^	0.16 ± 0.005^a^	0.21 ± 0.007^b^	0.14 ± 0.009^a^	0.17 ± 0.006^b^	0.15 ± 0.004^a^
Total				0.51	0.44	0.60	0.42	0.47	0.49
**Ketones**									
kt1	2-Octanone	1,312	MS,RI	0.07 ± 0.003^a^	0.08 ± 0.002^a^	0.06 ± 0.002^a^	0.09 ± 0.001^a^	0.09 ± 0.003^a^	0.10 ± 0.003^a^
kt2	2-Nonanone	1,390	MS,RI	1.96 ± 0.05^d^	1.77 ± 0.08^a^	1.83 ± 0.03^b^	1.91 ± 0.04^c^	1.90 ± 0.07^c^	1.96 ± 0.03^d^
Total				2.03	1.85	1.89	2.00	1.99	2.06
**Acids**									
ac1	Acetic acid	1,428	MS,RI	3.78 ± 0.19^d^	3.61 ± 0.21^b^	3.52 ± 0.12^a^	3.69 ± 0.11^c^	3.57 ± 0.17^a^	3.64 ± 0.12^b^
ac2	Butanoic acid	1,626	MS,RI	0.19 ± 0.01^b^	0.21 ± 0.04^c^	0.18 ± 0.008^a^	0.17 ± 0.03^a^	0.19 ± 0.009^b^	0.16 ± 0.01^a^
ac3	Hexanoic acid	1,855	MS,RI	0.08 ± 0.004^b^	0.09 ± 0.002^b^	0.06 ± 0.001^a^	0.08 ± 0.002^b^	0.04 ± 0.002^a^	0.07 ± 0.001^b^
ac4	Octanoic acid	2,070	MS,RI	0.12 ± 0.005^b^	0.13 ± 0.004^b^	0.09 ± 0.007^a^	0.11 ± 0.004^b^	0.08 ± 0.002^a^	0.14 ± 0.005^b^
Total				4.17	4.04	3.85	4.05	3.88	4.01
Sum				89.23	92.09	94.46	94.07	95.61	98.72

*Different letters in each row indicate significant differences (*p <* 0.05, *Duncan’s tests*) between different HQW. MS, compounds were identified by MS spectra. KI is the retention index calculated by the Kovats method. RI, compounds were identified by comparison to pure standard. RIL, compounds were identified by comparison with RI from http://webbook.nist.gov/. “Control” represents HQW without *FSI*; 0.1 *FSI*, 0.3 *FSI*, 0.5 *FSI*, 0.7 *FSI*, and 0.9 *FSI* represent HQW containing 0.1, 0.3. 0.5, 0.7, and 0.9 mg/mL *FSI*, respectively.*

As shown in Figure 4, esters were the most abundant aroma compounds, followed by alcohols and aldehydes. However, the total content of alcohols was the greatest, and thus responsible for the differentiation of total flavor compounds in different HQW. The addition of *FSI* significantly increased the total alcohol content in HQW. The highest alcohol content (62.36 mg/L) was observed in HQW containing 0.9 mg/L *FSI*, and this represented a 15.48% relative to the control. Among the detected alcohols, 3-methyl-1-butanol, 2-methyl-1-propanol, and 2-phenylethanol accounted for more than 95% of the total alcohols. Furthermore, the 3-methyl-1-butanol content gradually increased with the amount of *FSI* and was highest in HQW containing 0.9 mg/L *FSI*. The content of 2-phenylethanol in HQW containing *FSI* was significantly higher than that in the control, although the concentration of *FSI* had little effect on this parameter. Most of the detected esters in HQW were fatty-acid ethyl-esters (FAEE) (Figure 4 and Table 1)., comprising short-chain (SC, C_2_-C_5_), medium-chain (MC, C_6_-C_12_), and long-chain (LC, C_13_-C_18_) FAEE according to the chain length (Gao et al., 2018). As the *FSI* concentration was increased, the relative contents of SCFAEE and long-chain fatty acids ethyl esters (LCFAEE) in HQW also gradually increased. Compared to the control wine, the addition of 0.9 mg/L *FSI* increased the SCFAEE content by 20.95% in the resultant HQW, but only increased the LCFAEE content by 3.03% (Table 1). Benzaldehyde, phenylacetaldehyde, and furfural were identified as the key aldehydes in HQW (Yang et al., 2017; Liu Z. et al., 2020). The content of benzaldehyde determined the total content of aldehydes in each HQW (Table 1). However, no significant difference was observed between the total aldehyde contents in HQW with and without *FSI*, indicating that the addition of *FSI* had little effect on the aldehyde content of HQW during aging.

### Sensory Evaluation

**FIGURE 5:**
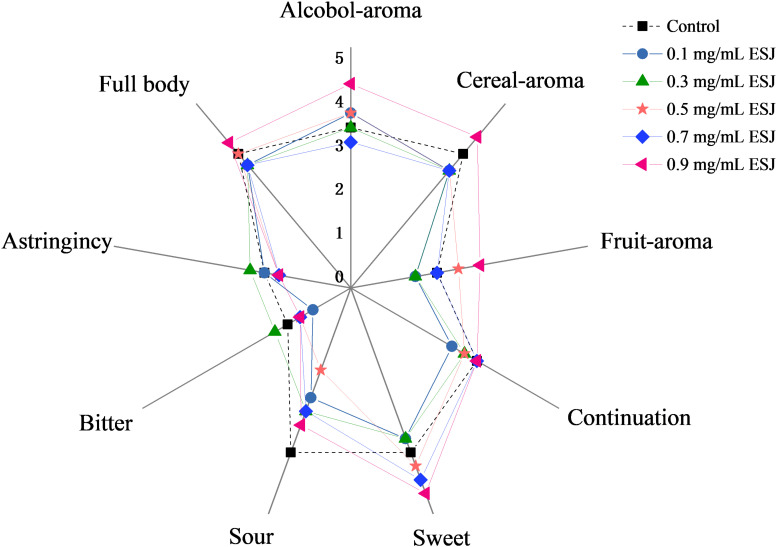
Scores of sensorial attributes for HQW treated with different concentrations of *FSI* after sterilization and aged for 160 days.

The aged HQW samples treated with different *FSI* concentrations after sterilization were subjected to a sensory evaluation. The mouthfeel characteristic of astringency, full-body, and aftertaste-continuation varied little in HQW containing different concentrations of *FSI* (Figure 5). In contrast, a significant difference in the aroma of alcohol, cereal, and fruity was observed. Different wines were perceived to have similar levels of sweet taste, however, the wines received significantly different scores for the taste of sour, despite a lack of significant differences in the total acidity. In summary, although the overall mouthfeel characteristics were similar between HQW samples with and without *FSI*, the concentration of *FSI* significantly affected the aroma characteristics of the wine. HQW treated with 0.9 mg/mL *FSI* received the highest scores for the total mouthfeel and aroma.

### Correlation Among Flavor Compounds, Sensory Attributes, and HQW With *FSI* Treatment

**FIGURE 6:**
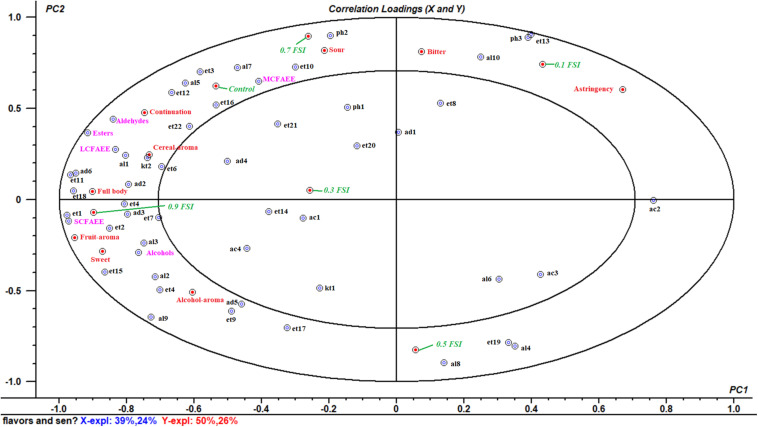
Correlataion loadings plot between the fifty-three volatile flavor compounds and nine sensory attributes in HQW containing different concentrations of *FSI* (The codes were defined in Table 1).

A multivariate analysis of the variance-PLSR model was performed to study the relationship between flavor compounds and sensory attributes in samples of HQW with and without *FSI*. A total of 53 identified flavor compounds were designed as the *X*-matrix, while nine sensory attributes and six wine samples were designed as *Y*-matrix (Figure 6). The two-factor model explained 63% of the variance in *X*-matrix and 76% of that in *Y*-matrix, indicating that this model satisfactorily explained the flavor compounds and sensory attributes. Except for nonanal, benzaldehyde, ethyl nonanoate, ethyl dodecanoate, ethyl stearate, ethyl (9E)-9-octadecenoate, acetic acid, hexanoic acid, octanoic acid, 2-octanone, 2-furanmethanol, and 2-methoxy-phenol, all of the sensory attributes and flavor compounds were located between the small and big ellipses, where the flavor compounds could be considered as correlated with sensory attributes (Xiao et al., 2014).

## Discussion

*Monascus* pigments, as the distinguished component in HQW, its photosensitivity characteristic significantly affects the sensory quality of the resulting wine. During the manufacturing processes of HQW, *Monascus* pigments may be degraded from the stage of Hong Qu soaking, wine fermentation, and sterilization. Though the pigment-preservation rate of HQW increased with *FSI* concentration, adding *FSI* after sterilization resulted in the greatest reduction of *Monascus* pigment loss in HQW, especially when added at a concentration of 0.9 mg/mL.

Typically, the ionization reaction occurs in a water solution of Monascus pigments under light, generating superoxide anions, hydroxyl radicals, and radicals originated by the side chain fracture (Feng et al., 2012). These radicals undergo an addition reaction with the double bonds in the pigment structure, which caused the disappearance of conjugated double bonds in *Monascus* pigments, and led to the loss of color (Mapari et al., 2009; Patakova, 2013). The antioxidant substances formed chemical bonds between its hydroxyl and the ketone group on the molecular structure of *Monascus* pigments, thereby stabilizing the structure of pigments (Kennedy et al., 1999). Reportedly, *FSI* is rich in antioxidant substances, such as rutin, quercetin, and chlorogenic acid, which might be related to its ability to preserve pigment under light (Hu et al., 2016). According to the HPLC analysis, the major antioxidant constituents of *FSI* were rutin and quercetin, and the content of rutin was significantly higher than quercetin (Figure 2). After UV irradiation, *FSI* exhibited better ability in the preservation of *Monascus* pigments than rutin or quercetin, suggesting that the combination of antioxidants enhanced the protective effect on *Monascus* pigments. However, the combination of multiple antioxidants greatly increases the cost. As a natural extraction product, *FSI* can replace the conventional antioxidants to protect natural pigments from light irradiation.

While adding *FSI* after HQW sterilization, it had little effect on the oenological properties, indicating that there was no need to change the production parameters due to the *FSI* treatment (Figure 3). However, *FSI* treatment contributed greatly to the flavor profiles of HQW. During the fermentation of HQW, the antioxidant constituents in *FSI* can prevent the growth and reproduction of microorganisms that contributed greatly to the stability of *Monascus* pigments (Tang et al., 2019). This may responsible for the reason that adding *FSI* after sterilization exhibited the best inhibitory effect on the *Monascus* pigment photodegradation. In addition, adding *FSI* during the Hong Qu soaking process or fermentation process will inhibit the metabolism of yeast, which generates a range of volatile flavor compounds and results in the specific flavor characteristic of HQW (Chen and Xu, 2012; Cai et al., 2018). This phenomenon affects its utilization as it reduces the sugar by microorganisms (including yeast), contributing to the formation of flavor compounds in HQW. After sterilization, the fresh HQW had an insufficient aroma and a rough taste, and a variety of chemical reactions such as oxidation, esterification, and hydrolysis reactions gradually occur (Jones et al., 2008; Tao et al., 2014). The added *FSI* may promote the chemical reactions among some flavor compounds, thereby affecting the flavor profiles of HQW. Generally, the flavor of Huangjiu is attributed to the combination of alcohols, esters, and aldehydes that make up the structural components of HQW aroma (Liu Z. et al., 2020). With *FSI* treatment, a significant difference in the total content of alcohols was observed in HQW samples containing different *FSI* concentrations, and *FSI* strongly enhanced the formation of 3-methyl-1-butanol and 2-methyl-1-propanol. In contrast, the total content of esters in HQW with *FSI* was generally lower than that in the control wine, except for HQW containing 0.7 and 0.9 mg/L *FSI* (Figure 4 and Table 1). At nearly all concentrations of *FSI*, the content of MCFAEE was significantly lower in treated HQW samples relative to the control; however, treatment with 0.9 mg/L *FSI* led to a higher content of MCFAEE relative to the control. These data suggested that the addition of *FSI* significantly promoted the accumulation of SCFAEE during HQW aging. As for aldehydes, the addition of FSI had little effect on the aldehyde content of HQW during aging. Thus, the *FSI* treatment improves the flavor profiles of HQW by affecting the production of alcohols and esters, especially 3-methyl-1-butanol, 2-methyl-1-propanol, and SCFAEE. Combining with the sensory evaluation (Figure 5), the treatment of 0.9 mg/mL *FSI* considerably highlighted the alcohol-aroma, cereal-aroma, and fruit-aroma in the aged HQW, consistent with the content of high alcohols and esters shown in Figure 4. The similar score of sweet taste in different HQW samples corresponded well with oenological properties, as the sweet attribute is partly related to the content of reducing sugar (Figure 3). The sour taste, which is generally related to the presence of organic acids (Dysvik et al., 2020), scored different levels suggesting that the *FSI* might have significantly affected the flavor-active organic acids in HQW.

As shown in Figure 6, the HQW samples treated with different *FSI* concentrations were located in different quadrants, suggesting that the concentration of *FSI* had a significant effect on the sensory characteristic of the resultant HQW. HQW treated with 0.7 mg/mL *FSI* exhibited a similar sensory profile as the control wine, and this profile was closely related to the presence of MCFAEE, 1-octen-3-ol, 3-methylthiopropanol, isoamyl acetate, diethyl succinate, ethyl tetradecanoate, and continuation. However, treatment with 0.7 mg/mL *FSI* was strongly correlated with a sour taste. Treatment with 0.1 mg/mL *FSI* was correlated to astringency mouthfeel, indicating that different *FSI* concentrations increased certain sensory attributes. Treatment with 0.9 mg/mL *FSI* correlated strongly with a full body and fruit aroma, and was favored by consumers. The sensory characteristic of the resultant HQW depended greatly on the concentration of *FSI*. The addition of 0.9 mg/mL *FSI* yielded a satisfactory result, consistent with the sensory evaluation (Figure 5). The key attributes of the fruit and alcohol aroma were closely related to the presence of SCFAEE and to that of 1-butanol and 2-phenylethanol, respectively. The high odor activity values of ethyl acetate, ethyl butanoate, ethyl hexanoate, and 2-phenylethanol might be responsible for the fruit aroma (Yang et al., 2018). Cereal-aroma was strongly correlated with the presence of LCFAEE, 2-nonanone, ethyl-2-hydroxypropanoate and 2-methyl-1-propanol, and different types of flavor compounds (esters, alcohols, aldehydes, and ketones) suggested a complex origin for this aroma attribute (Mo et al., 2010; Liu S. et al., 2019). Both a full body and fruit aroma were strongly correlated with the presence of SCFAEE. Combining with the result of flavor compounds that the SCAFEE content was significantly accumulated by adding *FSI* (Figure 4), it suggested that *FSI* may alter the aroma of HQW by enhancing the synthesis of SCFAEE. Thus, the addition of *FSI* to HQW after sterilization may be a novel alternative strategy for obtaining HQW with a high-quality flavor profile.

In this study, we demonstrated that the photodegradation of *Monascus* pigments in HQW could be reduced significantly by adding *FSI* to wine after sterilization. Specifically, *FSI* considerably enhanced the accumulation of alcohols and esters as the added amounts were increased, especially 3-methyl-1-butanol, 2-methyl-1-propanol, and SCFAEE, in HQW. Furthermore, *FSI* enhanced the alcohol, cereal, and fruit aromas in HQW. HQW treated with 0.9 mg/mL *FSI* received the highest scores for the mouthfeel and aroma. A PLSR analysis of the HQW samples revealed that a pleasing aroma was strongly related to the presence of SCFAEE, the accumulation of which was significantly enhanced by *FSI*. Therefore, we inferred that *FSI* might alter the aroma of HQW by enhancing the synthesis of SCFAEE. Taken together, our results suggest the considerable potential of *FSI* treatment as a strategy for reducing the photodegradation of *Monascus* pigments, thus improving the color stability, flavor profile, and sensory characteristics of the resulting HQW. Our work provides a new strategy for improving the pigment stability and sensory quality of HQW and other beverages containing photosensitive pigments. However, further studies are needed to explore the bioactive constituents of *FSI* in detail and the mechanisms by which *FSI* enhances the formation of SCFAEE.

## Data Availability Statement

The raw data supporting the conclusions of this article will be made available by the authors, without undue reservation.

## Ethics Statement

We obtained informed consent from participants in the sensory evaluation in accordance with the Code of Ethics of the World Medical Association (Declaration of Helsinki).

## Author Contributions

YY: experiment, investigation, software, data curation, and writing—original draft. YX: conceptualization, methodology, project administration, and writing—review and editing. XS: methodology, project administration, writing—review and editing. ZM: experiment, resources, and methodology. HQ: experiment, resources, methodology, and visualization. LT: conceptualization, software, and investigation. LA: supervision, writing—original draft, and writing—review and editing. All authors contributed to the article and approved the submitted version.

## Conflict of Interest

The authors declare that the research was conducted in the absence of any commercial or financial relationships that could be construed as a potential conflict of interest.

## Abbreviations

PLSR: partial least squares regression
SCFAEE: short-chain fatty acid ethyl esters
MCFAEE: medium-chain fatty acids ethyl esters
LCFAEE: long-chain fatty acids ethyl esters.
