# Supplementary material for: The Potential of Flos sophorae immaturus as a Pigment-Stabilizer to Improve the Monascus Pigments Preservation, Flavor Profiles, and Sensory Characteristic of Hong Qu Huangjiu
Source: Front Microbiol. 2021 May 20;12:678903. doi: 10.3389/fmicb.2021.678903 (PMC8174305; doi:10.3389/fmicb.2021.678903)
Supplement: Supplementary file 1 [file Table_1.DOC]

**Table S1** Definitions and reference scales of sensory attributes for HQW.

| Attributes | Definitions | Reference scales |
| --- | --- | --- |
| ***Aroma*** |  |  |
| Alcohol-aroma | Smell related to alcohol | Minimum: Water; Medium: 12.5% (w/v) Ethanol; Maximum: 25% (w/v) Ethanol. |
| Fruit-aroma | From fruit aroma (e.g. Banana) | Minimum: Water; Medium: 7.5 g Crushed banana/100 mL distilled water  Maximum: 15 g Crushed banana/100 mL distilled water |
| Cereal-aroma | Smell related to rice or barley | Minimum: Water; Medium: 2 g Crushed unpolished rice or barley/20 mL distilled water  Maximum: 4 g Crushed unpolished rice or barley/20 mL distilled water |
| ***Taste*** |  |  |
| Sweet | Sucrose as typical | Minimum: Drinking water; Medium: 3% (w/v) Sucrose; Maximum: 6% (w/v) Sucrose. |
| Sour | Vinegar taste | Minimum: Drinking water; Medium: 0.1 mL Vinegar/100 mL distilled water.  Maximum: 0.2 mL Vinegar/100 mL distilled water |
| Bitter | Caffeine as typical | Minimum: Drinking water; Medium: 0.05% Caffeine;  Maximum: 0.1% (w/v) Caffeine |
| ***Mouthfeel*** |  |  |
| Astringency | Dryness mouthfeel | Minimum: HQW aged for 6 years (Huizelong Wine Co., Ltd., Fujian Province, China);  Medium: Chinese rice wine aged for 3 years (Huizelong Wine Co., Ltd.)  Maximum: Young HQW (Huizelong Wine Co., Ltd.) |
| Continuation | Feeling of continuing taste | Minimum: Young HQW  Medium: HQW for 3 years  Maximum: HQW aged for 6 years |
| Full body | General feeling while tasting |
